# Long- versus short-interval follow-up after resection of hepatocellular carcinoma: a retrospective cohort study

**DOI:** 10.1186/s40880-018-0296-x

**Published:** 2018-05-21

**Authors:** Wei He, Yun Zheng, Ruhai Zou, Jingxian Shen, Junping Yang, Jiliang Qiu, Qiang Tao, Wenwu Liu, Zhiwen Yang, Yuanping Zhang, Binkui Li, Yunfei Yuan

**Affiliations:** 1 State Key Laboratory of Oncology in South China, Collaborative Innovation Center for Cancer Medicine, Sun Yat-Sen University Cancer Center, 651 Dongfeng Road East, Guangzhou, 510060 China; 2 Department of Hepatobiliary Oncology, Sun Yat-Sen University Cancer Center, Guangzhou, 510060 China; 3 Department of Ultrasound, Sun Yat-Sen University Cancer Center, Guangzhou, 510060 China; 4 Department of Medical Imaging, Sun Yat-Sen University Cancer Center, Guangzhou, 510060 China

**Keywords:** Hepatocellular carcinoma, Hepatectomy, Prognosis, Surveillance, Recurrence

## Abstract

**Background:**

Average postoperative follow-up intervals vary in patients undergoing hepatocellular carcinoma (HCC) resection because of limited evidence regarding the optimal interval. We aimed to compare the associations of long-versus short-interval follow-up with survival and recurrence in risk-stratified HCC patients.

**Methods:**

We performed a retrospective cohort study between 2007 and 2014. In total, 1227 patients treated by curative resection of Barcelona Clinic Liver Cancer stage A or B HCC were stratified as having a low (n = 865) or high (n = 362) risk of early recurrence (within the first 2 years after resection) based on prognostic factors identified by the least absolute shrinkage and selection operation algorithm. Patients were further classified into long-interval (every 4–6 months) and short-interval (every 2–4 months) follow-up subgroups based on follow-up within 2 years after resection (low risk, long vs. short: n = 390 vs. n = 475; high-risk, long vs. short: n = 149 vs. n = 213).

**Results:**

The short-interval follow-up did not prolong overall survival in either the low-risk (hazard ratio [HR] = 1.152; 95% confidence interval [CI] 0.720–1.843) or high-risk (HR = 1.213; 95% CI 0.702–2.094) patients. Early recurrence occurred in 401 patients. For high-risk patients, the short-interval follow-up subgroup exhibited smaller intrahepatic recurrence than did the long-interval group (2.6 vs. 3.5 cm, respectively, *P* = 0.045). However, no significant difference in the rate of Barcelona Clinic Liver Cancer stage 0/A recurrence was found between the long- and short-interval follow-up groups in either low- or high-risk patients (63.1% vs. 68.2%, respectively, *P* = 0.580; 31.3% vs. 41.5%, respectively, *P* = 0.280). The rate of curative intent treatment for recurrence (34.5% vs. 39.7%, respectively, *P* = 0.430; 14.6% vs. 20.3%, respectively, *P* = 0.388) was also similar between the follow-up groups for low- and high-risk patients.

**Conclusions:**

Shortening the postoperative follow-up interval from every 4–6 months to every 2–4 months within the first 2 years after resection did not increase the rate of curative intent treatment or prolong the overall survival of patients with Barcelona Clinic Liver Cancer stage A or B HCC.

## Introduction

Hepatocellular carcinoma (HCC) is one of the most common cancers and a leading global cause of cancer-related death, with China accounting for over half of the number of cases and deaths [1]. The high recurrence rate after curative hepatic resection for HCC, especially within the first 2 years after resection, remains a major challenge for long-term survival [2–4]. The primary purpose of postoperative follow-up is to identify recurrence at an early stage while curative intent treatment is still an option. The value of intensive postoperative follow-up has been studied in patients with colorectal cancer, breast cancer, non-small cell lung cancer and pancreatic cancer [5]. However, neither the European Association for the Study of the Liver and the European Organization for Research and Treatment of Cancer (EASL-EORTC) [6] nor the American Association for the Study of Liver Diseases (AASLD) have provided guidelines for the surveillance of HCC recurrence after hepatic resection [7]. The National Comprehensive Cancer Network [8] and the European Society for Medical Oncology (ESMO) [9] recommend that imaging (computed tomography [CT] or magnetic resonance imaging [MRI]) examination and serum alpha-fetoprotein (AFP) measurements be performed every 3–6 months for the first 2 years after resection and every 6–12 months thereafter. These recommendations are based on lower-level evidence (expert consensus) with limited supporting data, especially regarding the optimal time interval for each evaluation in the first 2 years after curative resection, which is when the majority of HCC recurs [10].

The lack of clear evidence regarding an optimal time interval for follow-up has resulted in clinical follow-up intervals ranging from 2 to 6 months [11]. A short-interval follow-up strategy may provide a better chance for early identification of recurrence, higher rate of curative intent treatment, and longer overall survival (OS), especially for patients considered high-risk for early recurrence. However, the hypothetical prognostic benefits of using a short interval for follow-up remain unknown, and the cost of this strategy has not been assessed [10].

In the present study, we compared the characteristics of early recurrence, the rate of curative intent treatment for recurrence, and OS between patients with long- and short follow-up intervals within the first 2 years after curative resection of HCC. We initially stratified patients into low- and high-risk groups for early recurrence based on their clinical characteristics and then classified them into short- and long-interval follow-up subgroups within each risk group according to postoperative surveillance time interval. We then compared the prognoses among the groups.

## Patients and methods

### Patient cohort

A prospective follow-up database and an electronic medical record system have been maintained at Sun Yat-sen University Cancer Center since 2002 to track and record all treated patients with HCC. In this retrospective cohort study, we reviewed the database and identified 2126 consecutive patients with Barcelona Clinic Liver Cancer stage (BCLC) A or B HCC who were initially treated with curative hepatic resection (tumor-negative resection margins) between January 2007 and December 2014. Patients with preoperative treatments, portal or hepatic vein invasion, or other malignant tumors were excluded. The resection procedure was performed as described in our previous article [12]. The study protocol conformed to the ethical guidelines of the Declaration of Helsinki, and the Ethics Committee of Sun Yat-sen University Cancer Center approved the study. Written informed consent was obtained before resection.

### Recurrence data collection

The diagnosis of recurrence was based on the results of imaging examinations (CT/MRI) and serum AFP tests. The date of recurrence was defined as the date of an initial positive result on imaging examination. In patients suspected to have HCC recurrence based on liver ultrasound, either abdominal CT or MRI was performed to confirm or exclude the diagnosis. Recurrence within the first 2 years after resection was defined as early recurrence.

Curative intent treatment for recurrence was defined as repeat hepatic resection, liver transplantation, or ablation as initial treatment for BCLC stage 0/A recurrence. Other treatments were regarded as noncurative intent treatments, including transarterial chemoembolization, molecularly-targeted therapy, and supportive treatment for advanced recurrence. The present study was censored on February 1, 2017.

### Risk groups

We hypothesized that patients at low- versus high-risk for early recurrence differentially benefit from short- versus long-interval follow-up. To ensure unbiased allocation when comparing the efficacy of follow-up, we stratified patients as low- or high-risk for early recurrence based on their risk score as in our previous study [13]. We first collected data on patient factors (gender, age, white blood cell count, red blood cell count, hemoglobin, platelet count, prothrombin time, albumin, total bilirubin, alanine aminotransferase, aspartate aminotransferase, albumin-bilirubin grade, AFP, etiological status, and cirrhosis); tumor factors (multiple tumors, tumor size, microvascular invasion, tumor cell differentiation, and tumor location); and resection factors (resection margin and operative blood loss). Then, we identified predictive factors for early recurrence and constructed a risk score model for the entire cohort using the least absolute shrinkage and selection operation (LASSO) algorithm with penalty parameter tuning conducted by tenfold cross-validation.

The prognostic factors for early recurrence selected by the LASSO algorithm were tumor size (cm), multiple tumors (1 = present, 0 = absent), microvascular invasion (MVI; 1 = present, 0 = absent), and nonhepatitis status (1 = no hepatitis, 0 = hepatitis B or C). We built a risk score model for early recurrence using a linear combination of weighted predictors as follows: Risk score = 0.380 * MVI + 0.309 * Multiple tumor + 0.073 * Tumor size − 0.119 * Nonhepatitis.

The optimal cutoff point value for risk stratification was determined using the maximally selected rank statistics of Maxstat (R statistical package, http://www.r-project.org, Vienna, Austria).

### Follow-up data collection

All patients received regular postoperative follow-up from the surgeon and/or the surveillance team at the hospital. Each follow-up consisted of a physical examination, serum AFP test, and at least one imaging examination (liver ultrasound, CT, or MRI). The first clinical visit was scheduled 3–4 weeks after resection for potential postoperative complications and was not considered follow-up for recurrence. We focused on follow-up within the first 2 years after resection, which is when most HCC recurs. The short- and long-interval follow-up protocols were defined as postoperative follow-up (CT/MR/ultrasound) every 2–4 months and every 4–6 months, respectively. For each patient, compliance with the follow-up plan was examined by comparing the observed and expected numbers of follow-up sessions by the time of the last follow-up or when recurrence was detected [14]. For example, by the 12th month after resection, the expected number of follow-up sessions was 2–3 (every 4–6 months) for the short-interval plan and 4–6 (every 2–4 months) for the long-interval plan. Patients with a follow-up interval of less than 2 months or more than 6 months were excluded, as these intervals were deemed irregular and likely to influence the outcome. Patients with recurrence detected within 3 months after resection were excluded from the follow-up grouping because this was considered a sign of nonradical resection.

### Statistical analysis

Continuous variables were compared using the independent samples t test and the Mann–Whitney U test, where appropriate. Binary and ordinal categorical variables were compared using the Chi squared test and the Kruskal–Wallis test, respectively. Recurrence-free survival (RFS) was defined as the time from date of resection to recurrence, and OS was defined as the time from resection to date of death with a censor date of last contact or June 1, 2017. Survival curves were constructed and compared using the Kaplan–Meier method and log-rank test, respectively. A Cox proportional hazards model was used to identify the prognostic factors for OS. Variables identified as significant on univariate analysis were entered into the Cox proportional hazards regression analysis to identify independent prognostic factors for survival. The proportional hazards assumption was verified by the Schoenfeld residual test and plots, and multicollinearity was evaluated using the variation inflation factor. Statistical analyses were performed using the R statistical package. *P* values less than 0.05 were considered statistically significant, and all tests were two-tailed.

## Results

### Stratification of patients by risk for early recurrence

We identified 2126 patients with BCLC stage A or B HCC who were initially treated with curative hepatic resection. Patients’ characteristics are shown in Table 1. The median follow-up time was 37.6 months (range: 22.0–61.4 months). In total, 39.6% (n = 842) of the patients developed recurrence (702 [83.4%] had early recurrence), and 15.6% (n = 332) of the patients died. For the entire cohort, the 2- and 5-year RFS rates were 65.8% and 51.4%, respectively.

**Table 1:** Baseline characteristics of 2126 patients who underwent resection for hepatocellular carcinoma stratified by risk of recurrence

Characteristics	Low-risk patients (*n* = 1425) (%)	High-risk patients (*n* = 701) (%)	*P*
Gender			0.509
Male	1244 (87.3)	619 (88.3)	
Female	181 (12.7)	82 (11.7)	
Age (years)	52 (17)	50 (18)	0.001
Tumor number			< 0.001
Solitary	137 (9.6)	293 (41.8)	
Multiple	1288 (90.4)	408 (58.2)	
Tumor size (cm)			< 0.001
> 5	246 (17.3)	532 (75.9)	
≤ 5	1179 (82.7)	169 (24.1)	
Tumor size (cm)	3.5 (2.2)	8 (4.8)	< 0.001
Tumor location			< 0.001
Central	389 (27.3)	54 (7.7)	
Subcapsular	1036 (72.7)	647 (92.3)	
Tumor differentiation			0.040
Poor	194 (13.6)	119 (17)	
Moderate and well	1231 (86.4)	582 (83)	
Tumor MVI			< 0.001
Yes	87 (6.1)	498 (71)	
No	1338 (93.9)	203 (29)	
BCLC stage			< 0.001
0/A	1380 (96.8)	482 (68.8)	
B	45 (3.2)	219 (31.2)	
Etiology			0.646
Non-hepatitis	137 (9.6)	70 (10)	
HBV	1267 (88.9)	624 (89)	
HCV	21 (1.5)	7 (1)	
WBC (10^9^/L)	5.8 (2.1)	6.3 (2.5)	< 0.001
RBC (10^9^/L)	4.75 (0.7)	4.8 (0.8)	0.010
Hemoglobin (g/L)	146.5 (17.5)	146 (21.0)	0.360
PLT (10^9^/L)	161 (77)	189.5 (92)	< 0.001
ALT (U/L)	35.7 (25.6)	38.4 (27.5)	0.002
AST (U/L)	31 (16.3)	39.6 (26.6)	< 0.001
ALB (g/L)	43 (4.6)	42.3 (4.8)	< 0.001
TBIL (μmol/L)	13.4 (6.6)	12.5 (6.2)	0.003
PT (s)	11.7 (1.3)	11.7 (1.3)	0.684
ALBI grade			< 0.001
I	1216 (85.3)	550 (78.5)	
II	209 (14.7)	151 (21.5)	
AFP (ng/mL)			< 0.001
> 200	468 (32.8)	332 (47.4)	
≤ 200	957 (67.2)	369 (52.6)	
Cirrhosis			0.092
Yes	1075 (75.4)	505 (72)	
No	350 (24.6)	196 (28)	
Resection margin (cm)	1 (1.5)	1 (1.0)	< 0.001
Operative blood loss (mL)			< 0.001
> 400	173 (12.1)	214 (30.5)	
≤ 400	1252 (87.9)	487 (69.5)	

Values are presented as the median (interquartile range) or n (%)

*MVI* microvascular invasion, *BCLC stage* Barcelona clinic liver cancer stage, *HBV* hepatitis B virus, *HCV* hepatitis C virus, *WBC* white blood cell, *RBC* red blood cell, *PLT* platelet, *ALT* alanine aminotransferase, *AST* aspartate aminotransferase, *ALB* albumin, *TBIL* total bilirubin, *PT* prothrombin time, *ALBI* albumin-bilirubin, *AFP* alpha-fetoprotein

**Fig. 1:**
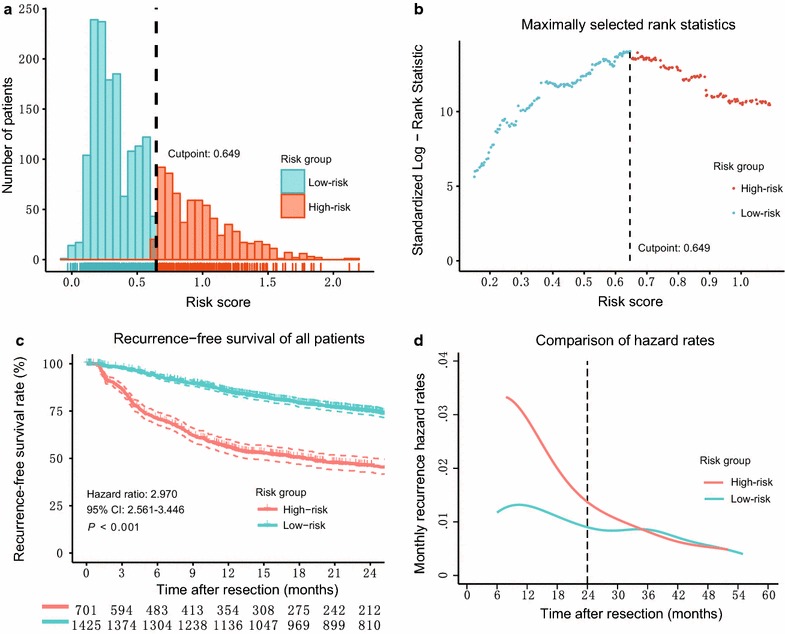
Risk scores for predicting early recurrence in 2126 patients who underwent resection for hepatocellular carcinoma. **a** Distributions of the risk scores calculated from the model using least absolute shrinkage and selection operation regression for early recurrence among the entire patient cohort. **b** The optimum cutoff value for the risk score was determined using the maximally selected rank statistics (cutoff point = 0.649, standardized log-rank statistic = 14.05). **c** The discriminative power of the risk score model for recurrence-free survival; 1425 (67.0%) and 701 (33.0%) patients were identified as low-risk and high-risk for early recurrence with a 2-year recurrence-free survival rate of 75.2% (95% confidence interval [CI] 72.9%–77.6%) and 46.4% (95% CI 42.6%–50.4%), respectively. Patients in the high-risk group showed worse recurrence-free survival (hazard ratio = 2.970; 95% CI 2.561–3.446; *P* < 0.001). **d** The high-risk group also showed a higher monthly recurrence hazard rate within the first 2 years of resection than did the low-risk group

The distributions of the risk scores and cutoff-point values for risk stratification are shown in Fig. 1. In total, 1425 patients (67.0%) with a risk score less than or equal to 0.649 were assigned to the low-risk group, and the remaining 701 patients (33.0%) were assigned to the high-risk group. Patients in the high-risk group had a worse RFS (HR = 2.970; 95% CI 2.561–3.446; *P* < 0.001) and a higher recurrence hazard rate within the first 2 years after resection than did those in the low-risk group (Fig. 1c and d).

### Patient follow-up

After reviewing the data from all 2126 patients, we found that 1227 (57.7%) patients had undergone regular follow-up every 2–6 months during the first 2 years after resection; 899 (42.2%) patients with irregular follow-up were excluded from the following analyses. The mean follow-up frequency was 2.7 and 4.4 times per year in the long- and short-interval follow-up groups, respectively. In total, 390 (45.1%) and 475 (54.9%) patients in the low-risk group underwent long- and short-interval follow-up, respectively, whereas 149 (41.2%) and 213 (58.8%) patients in the high-risk group underwent long- and short-interval follow-up, respectively. The proportion of high-risk patients undergoing short-interval follow-up did not significantly differ from the proportion of low-risk patients (*P* = 0.206). The baseline characteristics for the patients in each group appear in Table 2.

**Table 2:** Baseline characteristics of 1227 patients who underwent resection for hepatocellular carcinoma with regular follow-up stratified by risk of recurrence and follow-up interval

Characteristics	Low-risk patients			High-risk patients		
	Long interval	Short interval	*P*	Long interval	Short interval	*P*
	(*n* = 390)	(*n* = 475) (%)		(*n* = 149) (%)	(*n* = 213) (%)	
Gender			0.926			0.001
Male	344 (88.2)	418 (88)		123 (82.6)	199 (93.4)	
Female	46 (11.8)	57 (12.0)		26 (17.4)	14 (6.6)	
Age (years)	52 (14)	50 (19)	0.072	51 (17)	49 (17)	0.292
Tumor number			0.273			0.891
Solitary	359 (92.1)	427 (89.9)		92 (61.7)	130 (61)	
Multiple	31 (7.9)	48 (10.1)		57 (38.3)	83 (39)	
Tumor size (cm)			0.458			0.674
> 5	60 (15.4)	82 (17.3)		109 (73.2)	160 (75.1)	
≤ 5	330 (84.6)	393 (82.7)		40 (26.8)	53 (24.9)	
Tumor size (cm)	3.5 (2.0)	3.5 (2.3)	0.288	8 (5.0)	8 (4.8)	0.916
Tumor location			0.054			0.287
Central	96 (24.6)	145 (30.5)		16 (10.7)	16 (7.5)	
Subcapsular	294 (75.4)	330 (69.5)		133 (89.3)	197 (92.5)	
Tumor differentiation			0.393			0.761
Poor	45 (11.5)	64 (13.5)		20 (13.4)	31 (14.6)	
Moderate and well	345 (88.5)	411 (86.5)		129 (86.6)	182 (85.4)	
Tumor MVI			0.627			0.126
Yes	27 (6.9)	29 (6.1)		95 (63.8)	152 (71.4)	
No	363 (93.1)	446 (93.9)		54 (36.2)	61 (28.6)	
BCLC stage			0.604			0.238
0/A	380 (97.4)	460 (96.8)		110 (73.8)	145 (68.1)	
B	10 (2.6)	15 (3.2)		39 (26.2)	68 (31.9)	
Etiology			0.901			0.694
Non-hepatitis	37 (9.5)	47 (9.9)		18 (12.1)	22 (10.3)	
HBV	347 (89)	420 (88.4)		129 (86.6)	189 (88.7)	
HCV	6 (1.5)	8 (1.7)		2 (1.3)	2 (0.9)	
WBC (10^9^/L)	5.8 (1.9)	5.9 (2.3)	0.189	6.0 (2)	6.5 (2.8)	0.014
RBC (10^9^/L)	4.73 (0.7)	4.8 (0.7)	0.123	4.75 (0.7)	4.81 (0.7)	0.460
Hemoglobin (g/L)	147 (16.4)	146.7 (18)	0.922	144 (22)	145.9 (20.9)	0.392
PLT (10^9^/L)	161.3 (79.3)	162 (72)	0.526	192 (88)	189 (77)	0.643
ALT (U/L)	36.9 (27.2)	36.1 (23.7)	0.857	37 (26.7)	39.8 (29.6)	0.196
AST (U/L)	31.2 (16.8)	31.5 (14.8)	0.882	39.4 (25.4)	40.7 (25.2)	0.774
ALB (g/L)	42.8 (4.6)	43.1 (4.5)	0.083	42.2 (4.7)	43 (4.4)	0.074
TBIL (μmol/L)	12.9 (5.9)	13.4 (6.5)	0.129	12.2 (6.2)	12.7 (5.8)	0.502
PT (s)	11.7 (1.3)	11.6 (1.2)	0.590	11.6 (1.2)	11.6 (1.1)	0.615
ALBI grade			0.720			0.432
I	335 (85.9)	412 (86.7)		116 (77.9)	173 (81.2)	
II	55 (14.1)	63 (13.3)		33 (22.1)	40 (18.8)	
AFP (ng/mL)			0.289			0.905
> 200	111 (28.5)	151 (31.8)		66 (44.3)	93 (43.7)	
≤ 200	279 (71.5)	324 (68.2)		83 (55.7)	120 (56.3)	
Cirrhosis			0.957			0.296
Yes	288 (73.8)	350 (73.7)		95 (63.8)	147 (69)	
No	102 (26.2)	125 (26.3)		54 (36.2)	66 (31)	
Resection margin (cm)	1 (1.5)	1 (1.15)	0.526	1 (1.5)	1 (1.5)	0.894
Operative blood loss (ml)			0.691			0.173
> 400	45 (11.5)	59 (12.4)		52 (34.9)	60 (28.2)	
≤ 400	345 (88.5)	416 (87.6)		97 (65.1)	153 (71.8)	

Values are presented as the median (interquartile range) or n (%)

*MVI* microvascular invasion, *BCLC stage* Barcelona clinic liver cancer stage, *HBV* hepatitis B virus, *HCV* hepatitis C virus, *WBC* white blood cell, *RBC* red blood cell, *PLT* platelet, *ALT* alanine aminotransferase, *AST* aspartate aminotransferase, *ALB* albumin, *TBIL* total bilirubin, *PT* prothrombin time, *ALBI* albumin-bilirubin, *AFP* alpha-fetoprotein

### Comparison of early recurrence and treatment

Among the 1227 patients who were regularly followed-up, 401 patients were identified with early recurrence. High-risk patients were more likely to have large intrahepatic recurrence (2.84 ± 2.5 cm vs. 1.96 ± 1.29 cm, *P* < 0.001), multiple tumors (63.3% vs. 41.2%, *P* < 0.001), distant metastasis (29.5% vs. 9.4%, *P* < 0.001), and BCLC stage B/C recurrence (61.4% vs. 33.6%, *P* < 0.001) than low-risk patients, respectively.

The characteristics corresponding to early recurrence in the short- and long-interval follow-up groups are shown in Table 3. We found no difference in the size of intrahepatic recurrence between the long- and short-interval follow-up groups for low-risk patients (2.0 ± 1.3 cm vs. 1.9 ± 1.3 cm, respectively; *P* = 0.539); however, the high-risk patients in the short-interval follow-up group had smaller recurrent tumors than those in the long-interval group (2.6 ± 2.1 cm vs. 3.5 ± 3.3 cm, respectively; *P* = 0.045).

**Table 3:** Early recurrence and treatment details in the low- and high-risk patients

Characteristics	Low-risk patients			High-risk patients		
	Long interval	Short interval	*P*	Long interval	Short interval	*P*
	(*n* = 84) (%)	(*n *= 151) (%)		(*n* = 48) (%)	(*n* = 118) (%)	
Intrahepatic tumor size (cm)			0.539			0.045
Mean	2.0 ± 1.3	1.9 ± 1.3		3.5 ± 3.3	2.6 ± 2.1	
Median	1.7 (1.3)	1.6 (1.0)		2.5 (1.9)	2.1 (1.3)	
Recurrence number			0.394			0.383
1	44 (52.4)	86 (57.0)		13 (27.1)	38 (32.2)	
2	12 (14.3)	21 (13.9)		4 (8.3)	14 (11.9)	
3	4 (4.8)	5 (3.3)		0	9 (7.6)	
> 3	20 (23.8)	29 (19.2)		20 (41.7)	41 (34.7)	
Non-intrahepatic recurrence	4 (4.8)	10 (6.6)		11 (22.9)	16 (13.6)	
Recurrence location			0.686			0.755
Intrahepatic recurrence	77 (91.7)	136 (90.1)		33 (68.8)	84 (71.2)	
Distant metastasis	7 (8.3)	15 (9.9)		15 (31.3)	34 (28.8)	
Lung	5 (6)	8 (5.3)		12 (25.0)	27 (22.9)	
Bone	1 (1.2)	1 (0.7)		0	1 (0.8)	
Others	1 (1.2)	6 (4.0)		3 (6.3)	6 (5.1)	
Recurrence BCLC stage (A, B and C)			0.580			0.280
A	53 (63.1)	103 (68.2)		15 (31.3)	49 (41.5)	
B	22 (26.2)	28 (18.5)		15 (31.3)	31 (26.3)	
C	9 (10.7)	20 (13.2)		18 (37.5)	38 (32.2)	
Curative intent treatment for recurrence			0.430			0.388
Yes	29 (34.5)	60 (39.7)		7 (14.6)	24 (20.3)	
No	55 (65.5)	91 (60.3)		41 (85.4)	94 (79.7)	
Total treatment for recurrence (times)						
Resection	15	23		9	14	
Ablation	38	124		23	76	
LT	1	1		0	0	
TACE	62	134		26	114	
Radiotherapy	4	4		1	9	

Values are presented as the median (interquartile range), mean (standard deviation), or n (%)

*BCLC* Barcelona clinic liver cancer (stage), *LT* liver transplantation

There was no significant difference in the rate of solitary intrahepatic recurrence between short- versus long-interval follow-up groups for the low-risk (52.4% vs. 57.0%, respectively; *P* = 0.394) and high-risk patients (27.1% vs. 32.3%, respectively; *P* = 0.383). There was also no difference in the rate of distant metastatic recurrence between the long- and short-interval follow-up groups for the low-risk (8.3% vs. 9.9%, respectively; *P* = 0.686) and high-risk patients (31.3% vs. 28.8%, respectively; *P* = 0.755).

We classified recurrence according to the BCLC staging system, which accounted for the size, number, and location of each recurrence. There was no significant difference in the rate of BCLC stage 0/A recurrence between the long- and short-interval follow-up groups for the low-risk (63.1% vs. 68.2%, respectively; *P* = 0.580) and high-risk patients (31.3% vs. 41.5%, respectively; *P* = 0.280).

Following early recurrence, the rates of curative intent treatment for recurrence were similar between the long- and short-interval follow-up groups for both the low-risk (34.5% vs. 39.7%, respectively; *P* = 0.430) and high-risk (14.6% vs. 20.3%, respectively; *P* = 0.388) patients.

### Comparison of survival

**Fig. 2:**
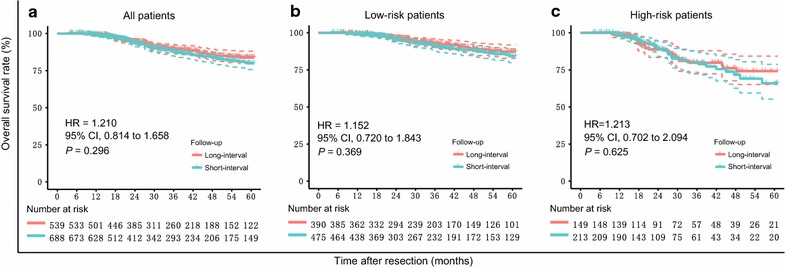
Survival curves and risk tables for 1227 patients by long- and short-interval follow-up. **a** Overall survival (OS) for all 1227 patients. The 3- and 5-year OS rates were 89.5% and 83.8%, respectively, in the long-interval group and 88.0% and 79.9%, respectively, in the short-interval follow-up group. **b** OS for the 855 low-risk patients. The 3- and 5-year OS rates were 93.0% and 87.2%, respectively, in the long-interval group and 91.0% and 84.3%, respectively, in the short-interval follow-up group. **c** OS for the 362 high-risk patients. The 3- and 5-year OS rates were 79.7% and 74.1%, respectively, in the long-interval group and 80.1% and 65.9%, respectively, in the short-interval follow-up group. Solid curves = survival curves; dashed curves = 95% confidence intervals

For the 1227 patients who were regularly followed-up, the median follow-up time was 38.7 months (range: 24.3–61.4 months) and 34.3 months (range: 18.8–61.9 months) for the long- and short-interval follow-up groups, respectively. Both groups had a similar OS (*P* = 0.296; Fig. 2a). The Cox proportional hazards model identified the independent adverse prognostic predictors for OS as multiple tumors (HR = 2.058; 95% CI 1.386–3.057; *P* < 0.001); tumor size > 5 cm (HR = 1.584; 95% CI 1.079–2.325; *P* = 0.019); and MVI (HR = 1.703; 95% CI 1.301–1.965; *P* = 0.008) (Table 4). After adjusting for these key confounding factors, no difference in survival was found between the long- and short-interval follow-up groups (HR = 1.210; 95% CI 0.814–1.658; *P* = 0.408). We found no significant difference in OS between the two follow-up groups for the low-risk (*P* = 0.369, Fig. 2b) and high-risk (*P* = 0.625, Fig. 2c) patients.

**Table 4:** Univariate and multivariate analysis of overall survival in all 1227 patients

Variables	Univariate analysis		Multivariate analysis		
	Wald Chi square	*P*	HR	95% CI	*P*
Gender (male:female)	0.33	0.567			
Age (year) (> 60: ≤ 60)	0.04	0.834			
PLT ((10^9^/L)) (≤ 100: > 100)	0.11	0.742			
ALB (g/L) (≤ 35: > 35)	0.04	0.848			
TBIL (μmol/L) (> 17.1: ≤ 17.1)	3.48	0.062			
PT (s) (> 16: ≤ 16)	0	0.993			
AFP (ng/mL) (> 200: ≤ 200)	0.53	0.468			
Hepatitis (yes:no)	1.53	0.216			
Cirrhosis (yes:no)	0.66	0.416			
Tumor number (multiple:solitary)	18.8	< 0.001	2.058	1.386–3.057	< 0.001
Tumor size (cm) (> 5: ≤ 5)	14.4	< 0.001	1.584	1.079–2.325	0.019
MVI (yes: no)	8.1	0.006	1.703	1.301–1.965	0.008
Tumor differentiation (poor:others)	1.22	0.269			
Tumor location (non-subcapsular:subcapsular)	2.79	0.095			
Resection margin (cm) (≤ 1: > 1)	0.97	0.323			
Hemorrhage (mL) (> 400: ≤ 400)	7.6	0.006	1.361	0.896–2.066	0.149
ALBI grade (II:I)	3.46	0.063			
Follow-up interval (short:long)	1.03	0.311			

*PLT* platelet, *ALB* albumin, *TBIL* total bilirubin, *PT* prothrombin time, *AFP* alpha-fetoprotein, *MVI* microvascular invasion, *ALBI* albumin-bilirubin

**Fig. 3:**
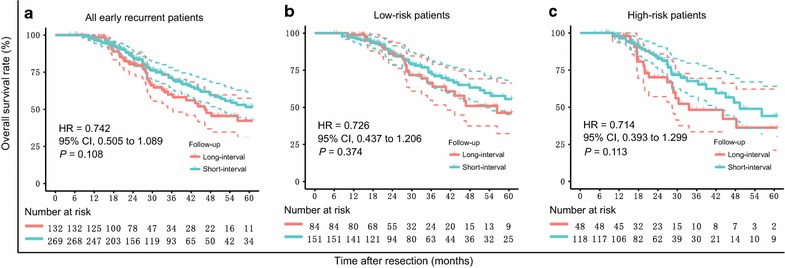
Survival curves and risk tables of for early recurrence patients by long- and short-interval follow-up. **a** Overall survival (OS) for the 401 early recurrence patients. The 3- and 5-year OS rates were 59.9% and 42.2%, respectively, in the long-interval group and 71.1% and 51.5%, respectively, in the short-interval follow-up group. **b** OS for the 235 low-risk patients. The 3- and 5-year OS rates were 66.6% and 46.3%, respectively, in the long-interval group and 73.5% and 55.6%, respectively, in the short-interval follow-up group. **c** OS for the 166 high-risk patients. The 3- and 5-year OS rates were 48.3% and 36.2%, respectively, in the long-interval group and 67.6% and 44.1%, respectively, in the short-interval follow-up group. Solid curves = survival curves; dashed curves = 95% confidence intervals

For the 401 patients with early recurrence, the median follow-up time was 32.4 months (range: 23.4–52.9 months) and 33.1 months (range: 20.2–55.0 months) for the long- and short-interval follow-up groups, respectively. Both groups had a similar OS (*P* = 0.108; Fig. 3a), and after adjustment, we found no difference in survival (HR = 0.742; 95% CI 0.505–1.089; *P* = 0.128). We also found no significant difference in OS between the follow-up groups for the low-risk (*P* = 0.374, Fig. 3b) and high-risk (*P* = 0.113, Fig. 3c) patients.

## Discussion

Our study provides evidence regarding the efficacy of short- and long-interval follow-up plans within the first 2 years after curative resection for HCC. We found no significant benefit from short-interval follow-up for patients regarding recurrence stage, curative intent treatment rate for recurrence, or OS.

The central goal of postoperative surveillance is to prolong OS by identifying early recurrences while they are still amenable to curative intent treatments. However, our study suggests that neither low-risk nor high-risk patients benefit from short-interval follow-up for either curative intent treatment rate or OS. There are varying reasons for the similar outcomes among the risk-stratified patients.

For low-risk patients, the short-interval follow-up did not identify recurrence at an early phase regarding recurrence size, number, location, or stage. Thus, low-risk patients may not benefit significantly from short-interval follow-up because 66.4% of recurrences were identified at an early stage (BCLC stage 0/A) when they had less malignant characteristics and a slow growth rate. As a result, we found no significant advantage with short-interval follow-up for low-risk patients regarding curative intent treatment rate or OS.

For high-risk patients, although intrahepatic recurrences could be identified at a smaller size using short-interval follow-up, 61.4% of recurrences were identified at an advanced stage (BCLC stage B/C) when they already exhibited extrahepatic metastasis, multiple tumors, or portal and hepatic vein invasion. As a result, for high-risk patients, a decrease in tumor size did not increase the rate of curative intent treatment for recurrence. One trial comparing the efficacies of 3- and 6-month screening intervals for HCC in patients with compensated cirrhosis also found that a short-interval follow-up was associated with smaller lesions but not with an increased rate of liver transplantation or better survival [15].

Intensive surveillance has been reported to improve survival in patients with breast [16] and colorectal [17] cancer after curative treatment. This improvement is because intensive surveillance can identify recurrence early while curative intent treatment is still an option. Also, 16%–33% of patients with isolated but initially unresectable hepatic metastases show sufficient response to conversion chemotherapy, permitting subsequent curative intent resection [18, 19]. Recently, the prognosis of patients with advanced colorectal [20] or breast [21] cancer has improved following the introduction of effective chemotherapy and molecularly-targeted therapy; therefore, the benefit of intensive surveillance on survival is significant in these patients and is associated with a favorable prognosis [5]. However, this situation may differ in patients with recurrent non-small cell lung cancer [22] or pancreatic cancer [23] for which the benefit of treatment for recurrence is minimal and the role of intensive postoperative follow-up is limited [5]. The situation is similar in patients with HCC because of the typically aggressive biological characteristics of the cancer and the likelihood of underlying chronic liver disease, especially in high-risk patients. Effective treatments are limited for high-risk patients with advanced recurrence for two reasons. First, HCC is considered a relatively chemotherapy-refractory tumor because of high expression of drug resistance genes [24–27], and patients with underlying liver dysfunction do not tolerate chemotherapy well. Second, the molecular pathogenesis of HCC is poorly understood, and only sorafenib monotherapy is approved as a systemic treatment for advanced HCC. However, the actual survival gain is less than 3 months in both Western [28] and Asian populations [29]. As a result, short-interval follow-up for high-risk patients simply detects earlier phases of potentially advanced recurrence, and thus, noncurative treatment might not significantly benefit OS.

To our knowledge, no study has evaluated the effect of intensive surveillance on patient quality of life following HCC resection. Another concern is physical harm from unnecessary radiation exposure from CT scanning. A recent study showed that extending the interval of CT scanning from 3 to 4 months reduces radiation exposure without compromising the rate of detection for HCC recurrence [30]. Also, the cost of examinations within the first 2 years after resection per patient in the short-interval follow-up was 23.6% higher than that in the long-interval group in our study (data not shown). The total healthcare cost saved by extending the follow-up interval would be significant in China, where approximately 466,100 new cases of liver cancer are diagnosed per year [31].

The present study has several limitations. First, we did not validate the risk score model of early recurrence using an independent center. However, the primary aim of the risk score was not to establish a predictive model but rather to stratify patients into low- and high-risk groups of early recurrence for further comparison. Second, we focused only on the effectiveness of different follow-up strategies within the first 2 years after resection. The value of different strategies thereafter remains unknown. Third, our results are based on a single-center study, and validation through a large multicenter study is necessary. We suggest that a multicenter, randomized controlled trial be performed to further investigate this issue.

In conclusion, the results of the present study suggest that shortening the follow-up interval from every 4–6 months to every 2–4 months within the first 2 years after curative resection for BCLC stage A or B HCC does not significantly improve patient prognosis regarding early identification of recurrence, the rate of curative intent treatment for recurrence, or OS.

## Abbreviations

HCC: hepatocellular carcinoma
AFP: alpha-fetoprotein
RFS: recurrence-free survival
OS: overall survival
MVI: microvascular invasion
BCLC: Barcelona clinic liver cancer stage
LASSO: least absolute shrinkage and selection operation
